# Comprehensive biocompatibility profiling of human pancreas-derived biomaterial

**DOI:** 10.3389/fbioe.2025.1518665

**Published:** 2025-04-25

**Authors:** Amish Asthana, Amanda Gallego, Quentin Perrier, Tamara Lozano, Lori N. Byers, Jun Ho-Heo, Wonwoo Jeong, Riccardo Tamburrini, Arunkumar Rengaraj, Deborah Chaimov, Alice Tomei, Christopher A. Fraker, Sang Jin Lee, Giuseppe Orlando

**Affiliations:** ^1^ Department of Surgery, Wake Forest Baptist Medical Center, Medical Center Boulevard, Winston-Salem, NC, United States; ^2^ Wake Forest Institute for Regenerative Medicine, Winston-Salem, NC, United States; ^3^ Wake Forest School of Medicine, Medical Center Boulevard, Winston-Salem, NC, United States; ^4^ Nanoimmunotech, Vigo, Spain; ^5^ Univ. Grenoble Alpes, Pharmacy Department, Grenoble Alpes University Hospital, Grenoble, France; ^6^ Diabetes Research Institute, University of Miami, Miami, FL, United States

**Keywords:** human pancreas, extracellular matrix, decellularization, biocompatibility, islet transplantation, biomimetic, 3D bioprinting, vascularization

## Abstract

**Introduction:**

The importance of the extracellular matrix (ECM) to pancreatic islets has been clearly demonstrated, as isolated islets grown in culture or transplanted, quickly lose viability and function after their matrix associations have been stripped away during the isolation process. Therefore, recapitulating the islet niche is a critical objective to move the field of islet transplantation forward.

**Methods:**

As a first step to recreating the islet microenvironment, we have recently developed a detergent-free decellularization method to obtain a decellularized solubilized ECM (dsECM) powder from human pancreas. We have also shown that this gentler method (compared to traditional detergent-based methods) allows for thorough preservation of the molecular fingerprint of the innate organ. Furthermore, incorporation of dsECM in alginate-microencapsulated human islets, showed a significant increase in insulin secretion, compared to both free and alginate-only encapsulated islets. However, it is also essential to test the interaction of dsECM with multiple cell types to establish its safety for transplantation.

**Results and discussion:**

Herein, we present a comprehensive *in vitro* evaluation of the cytotoxicity, hemocompatibility and immunocompatibility of dsECM to establish a concentration range where it deemed safe and biocompatible. Furthermore, dsECM-based bioinks were coaxially bioprinted and the resulting construct's biocompatibility and vascularization potential were also evaluated *in vivo*.

## 1 Introduction

The extracellular matrix (ECM) is a 3D structural framework of proteins and poly-saccharides that provides a spatial, biophysical and biochemical microenvironment to the cells (Asthana et al., 2021; Tamburrini et al., 2020). In the context of human pancreas, the islets have a very extensive network of ECM molecules. However, during the harsh and complex isolation process (Perrier et al., 2021), islets undergo enzymatic digestion that can result in irreversible ECM damage. As ECM-islet signaling is critical for islet function, destruction of the islet niche and ECM is deemed to contribute to the limited graft survival observed in clinical islet transplantation (Daoud et al., 2010; Irving-Rodgers et al., 2014; Kuehn et al., 2014; Tomei et al., 2014; Miao et al., 2013). Therefore, research efforts have been focussed on the recapitulation of the islet niche, which is a critical objective to move the field of islet transplantation forward. Since the very first report on the ability of the ECM to enhance rat islet cells attachment, proliferation and long-term culture maintenance (Thivolet et al., 1985), a large body of literature has provided strong evidence that restoration of the ECM in human islets can enhance islet function (Salvatori et al., 2014; Mirmalek-Sani et al., 2013; Peloso et al., 2016; Llacua et al., 2018). For instance, recent data has shown that the incorporation of ECM within capsules enhances insulin producing cells function and facilitates euglycemia with a significantly lower than usual number of insulin producing cells (Chaimov et al., 2017), while incorporation of critical components of the pancreatic ECM can significantly improve the endocrine function of β-cells (Hadavi et al., 2019). As both islets and ECM are very complex entities, it is often difficult to pinpoint specifically which islet cell type or which ECM component is responsible for these outcomes. So, rather than using single matrix components, using decellularized ECM could be more physiologically relevant.

Traditional organ decellularization techniques use detergents or surfactants to dissolve the cell membrane (Napierala et al., 2017; Elebring et al., 2017; Wan et al., 2017). However, detergents can also be potentially cytotoxic and possess the ability to trigger the immune system (Playfair and De Souza, 1986). Several studies have demonstrated that despite repeated washing steps, it is not possible to entirely remove all traces of detergent/surfactant from the treated biological material (Caamaño et al., 2009; Cebotari et al., 2010). Residual detergent can also alter the biophysical properties of ECM elastin fibers, affecting their mechanical strength and resulting in subsequent structural degradation (Guantieri et al., 1983; Kagan et al., 1972; Jordan et al., 1974). In order to overcome this limitation, our group recently developed a detergent-free Deionized (DI) water-based protocol for the decellularization of human pancreas. This decellularized ECM was solubilized with pepsin in hydrochloric acid (pepsin-HCl), followed by neutralization. The neutralized solution was then centrifuged and the supernatant was lyophilized to produce a growth factor-rich decellularized solubilized (dsECM) powder (Asthana et al., 2021; Tamburrini et al., 2020; Asthana et al., 2023). We had previously published a comprehensive Mass Spectrometry (MS)- and Enzyme-Linked Immunosorbent Assay (ELISA)-based proteomic characterization of our pancreatic dsECM, demonstrating that it retains at least 33.3% of native ECM proteins after solubilization including multiple crucial growth factors (22 in dsECM vs. 52 in native) and cytokines (40 in dsECM vs. 64 in native) that might promote the viability and function of pancreatic islets (Asthana et al., 2021). Fibrillar collagens were highly conserved in the dsECM. This study (Asthana et al., 2021) also confirmed that the solubilization process did not alter its composition as the protein distribution was similar between native and dsECM.

The dsECM was found to be enriched for protein families critical in the regulation of pancreatic beta cell differentiation/proliferation and pancreatic developmental processes. We had also incorporated dsECM in alginate (1.5% Ultra-Pure Low Viscosity Mannuronate; UP-LVM)-microencapsulated human islets, which preserved islet functionality during long-term (58 days) *in vitro* culture (Asthana et al., 2023). This further demonstrate that despite the solubilization process, the dsECM enhanced insulin secretion in encapsulated islets and supported cellular viability, strongly suggesting that sufficient bioactivity of the dsECM was maintained. Although we did not observe any adverse effects on islet viability, we envisioned a dsECM-supplemented islet construct for *in vivo* implantation that not only supports islet function, but also induces vascularization to enhance graft integration. Therefore, it is essential to test the interaction of dsECM with multiple cell types to establish its safety for transplantation. Considering this ultimate objective, a comprehensive evaluation of the cytotoxicity, hemocompatibility and immunocompatibility of dsECM was performed according to the American and European regulations with respect to nanosafety, following ISO10993 standards. Furthermore, we have also developed dsECM-based bioinks for 3D bioprinting and automated scale-up of construct biofabrication for therapy as well as model development. This also includes preliminary *in vivo* studies to assess the biocompatibility and vascularization potential of the resulting constructs.

## 2 Materials and methods

In accordance with internationally recognized standards (ISO 10993 and ASTM protocols) all methodological procedures, including statistical analyses, were selected to ensure the relevance and reliability of the biocompatibility assessments. Detailed descriptions of the methodological criteria applied throughout the study are provided in the Supplementary Material (Supplementary Table S1) to facilitate full transparency and reproducibility of the results.

### 2.1 Pancreas decellularization and solubilization

Human pancreases procured from deceased donors and allocated for research purposes were obtained under an institutionally-approved protocol #IRB00028826 (Wake Forest University Health Sciences) and stored in sterile conditions at −20°C. Pancreases were collected from adult donors with a BMI (Body mass index) < 30, with no known history of diabetes. Before decellularization, the frozen pancreases were thawed overnight at 4°C, dissected in approximately 1 cm^3^ cubes and then washed in 1,000 mL of deionized water containing 50 mL of betadine and Penicillin Streptomycin solution for 15min. Decellularization was accomplished using our non-detergent-based protocol as recently described (Tamburrini et al., 2020). Thereafter, the resulting decellularized cubes were frozen at −80°C, lyophilized, and cryomilled to obtain a fine dECM powder. This dECM powder was then solubilized with pepsin-HCl (0.01 M) for 48 h at room temperature and neutralized with 0.1N NaOH to obtain a pH of 7.4 at 4°C. The neutralized solution was then centrifuged at 10,000 × g, and the supernatant was lyophilized (Labconco) to produce the solubilized growth factor-rich dsECM powder.

Each pancreas was processed individually and then the milled dsECM powder was tested for DNA (<50 ng/mg of dry tissue). Thereafter, dsECM from five pancreases was pooled together to form a batch that was used for further experiments.

### 2.2 Cell proliferation-viability

Different cell lines were chosen in order to evaluate the cytotoxicity of the dsECM. HEK293 derived from human embryonic kidney cells, A549 as adenocarcinoma human alveolar cell line, and Jurkat cell line as a model of human T lymphocyte. Cells were cultured in Roswell Park Memorial Institute (RPMI) 1,640 Medium supplemented with 25 mM HEPES (Gibco), 10% (v/v) fetal bovine serum (Gibco, #A5256701), 100 U/mL penicillin and 100 µg/mL streptomycin (Gibco) at 37°C and 5% CO_2_. At growth phase of each cell line (based on previous laboratory experience working with these cell), regular medium was replaced with medium containing dsECM at concentrations of 0.125, 0.25, 0.5, 1.0 or 2.0 mg/mL. The cell proliferation was measured by the MTS assay (Promega) after 24 h of incubation. The percentage of cell viability was normalized to cells without dsECM. Triton x-100 was used as a positive control for cytotoxicity. Data was analyzed using one-way ANOVA with Dunnett’s test for multiple comparisons.

### 2.3 Apoptosis assay

Jurkat cells were cultured for 6 h in medium containing dsECM at concentrations of 0.125, 0.25, 0.5, 1.0 or 2.0 mg/mL. Early and late apoptosis, necrotic and viable cells were assessed using the combination of Annexin V/Propidium Iodide with a commercial apoptosis detection kit (Immunostep) followed by Flow Cytometry. Camptothecin (Sigma Aldrich) at 4 µg/mL was used as a positive control for apoptosis due to its well-known mechanism for blocking DNA replication (Liu et al., 2000). The percentage of each population was analysed by FlowJo™ (software for flow cytometry analysis) and represented for comparison and analysis.

### 2.4 Reactive oxygen species (ROS) quantification assay

The content of intracellular reactive oxygen species (ROS) was analyzed by measuring the oxidation of the probe DCFH-DA (2′, 7-dichlorofluorescein diacetate; Invitrogen). Jurkat and A549 cells were incubated for 4 h in medium containing dsECM at concentration of 0.125, 0.25, 0.5, 1.0 or 2.0 mg/mL. Following the incubation with DCFH-DA, oxidized in the presence of ROS, the fluorescent compound obtained was assessed by flow cytometry. As a positive control, PMA (phorbol myristate acetate, Abcam) at 10 μM was used. The fluorescence increase of 1.4-fold with respect to the negative control was considered a positive result.

### 2.5 Hemolysis

Human blood sample collection for hemocompatibility assays, complement system assay and peripheral blood mononuclear cells (PBMCs) isolation, were approved by the ethical committee of the Xunta de Galicia (Authorization No 2018/101). Hemocompatibility was determined in order to evaluate the interaction of the soluble pancreatic dsECM with red blood cells, platelets and coagulation factors. The hemolytic potential was quantified by colorimetric determination of hemoglobin in whole-blood when blood was exposed to dsECM at concentrations of 0.1 and 1.0 mg/mL for 2 h. A hemoglobin standard was used to build up a standard curve. In addition, commercial quality control samples were used at low, medium, and high concentration of hemoglobin to monitor assay performance (Teco Diagnostics). Blood from two human donors was pooled together. Triton X-100 was used to induce hemolysis for the positive control and untreated samples were used as negative control.

### 2.6 Platelet activation

Platelet activation was determined with the assessment of CD62P, which is a platelet-specific selectin protein, expressed on the surface of the activated platelets. The expression of CD62P was assessed with flow cytometry using a FITC Mouse Anti-Human CD62P antibody (BD Biosciences). dsECM was tested at concentrations of 0.125, 0.5 and 1.0 mg/mL and 15 min of exposure. Adenosine diphosphate (ADP) at 20 µM was used as positive control. ADP was also added to each tested sample in order to assess a potential inhibitory effect of dsECM. A positive result was considered upon increase of fluorescence of 2-fold with respect to the negative control. Blood from two human donors was pooled together.

### 2.7 Thrombogenecity and coagulation

The possible effects of dsECM in the coagulation cascade were determined by assessing changes in the intrinsic, extrinsic, and common coagulation pathways due to the binding or depletion of coagulation factors with the biomaterial. These changes were evaluated by measuring the time necessary (in seconds) for clot formation. Briefly, dsECM at concentrations of 0.1, 0.5 and 1.0 mg/mL was incubated with a pool of human plasma from at least three donors and the coagulation pathways were evaluated following the manufacturer´s protocol (Diagnostica Stago, more information is available in Supplementary Table S1). Clot formation was detected by a viscosity-based detection system using a hemostasis analyser (STart^®^, Stago). Normal and pathological (provided in the kit) plasma were used as internal controls. The pooled plasma only with the diluent solution was used as negative control.

### 2.8 Complement system activation assay

The complement system activation was assessed by analysing the degradation of C3 factor by western blot. A pool of human plasma from three donors was incubated with dsECM at concentrations of 0.1 and 1.0 mg/mL for 1 h and the supernatant was analysed by western blot. Zymosan A (Sigma Aldrich) at 1.0 mg/mL from *Saccharomyces cerevisiae* was used as positive control and EDTA (Sigma Aldrich) at 10 mM was used as a reversion reaction control. The immunoblot membrane was revealed with an antibody specific for C3b (Abcam). The intensity of the band corresponding to C3b fraction were compared respect to basal level of C3b (negative control) using ImageLab software (version 3.0). A positive result is considered upon increase of > 2-fold respect to the negative control.

### 2.9 Lymphocyte proliferation assay

Human PBMCs were isolated with Ficoll-Paque™ PLUS Media (GE Healthcare) and stained with CFSE (carboxyfluorescein diacetate succinimidyl ester; Invitrogen). Then PBMCs were cultured (RPMI 1640 medium supplemented with 10% human AB serum) with and without dsECM at 0.1 mg/mL in culture medium and allowed to proliferate for 7 days. The proliferation rate was determined by flow cytometry. Phytohemagglutinin (PHA-M; Roche) at 10 µg/mL was used as a positive control, as a recognized polyclonal T-cell mitogen and the negative control consisted of PBMCs cultured under identical conditions but without dsECM. A pool of human blood from 3 donors was used.

### 2.10 Caspase-1 activation assay

Caspase-1 activation assay was performed using a fluorescent inhibitor probe to label active caspase-1 enzyme in living cells. For this purpose, the FAM FLICA Caspase-1 Assay Kit (immunochemistry) was used. THP-1 cell line was selected to test inflammasome stimulation due to the high basal level of pro-caspase-1. Fluorescent signal was observed with Camptothecin (Sigma Aldrich) at 4 µg/mL as positive control. dsECM was tested at 0.1 mg/mL in cell culture medium and the detection of caspase-1 was performed by flow cytometry after 5 h of incubation. The negative control consisted of THP-1 cells cultured under identical conditions but without the dsECM compound.

### 2.11 Cytokine production assay

Potential inflammatory induction of dsECM was assessed by measuring the level of cytokines produced by PBMCs. PBMCs were isolated from blood samples pooled from three healthy donors. PBMCs were isolated using Ficoll-Paque™ PLUS Media (GE Healthcare) and incubated for 24 h in the RPMI 1640 culture medium containing either 0.1 or 1.0 mg/mL dsECM. PHA-M (10 µg/mL) (Roche) and LPS (1 µg/mL) (Invivogen) were used as positive controls. After incubation, the supernatant was collected for cytokine quantification by Luminex using a magnetic bead panel (Merck). The concentration of each cytokine (IL-1β, IL-6, TNF-α, IL-4, IL-13, MCP-1, IP-10, MIP-1α, IL-8, RANTES, VEGF, IL-2, IL-10, TNF-β, IFN-Gamma, IL-17a, GM-CSF, IL-12p70, IL-5, IL-1α) was determined by the individual standard curve. All the cytokine concentrations were normalized to negative (untreated) control. A positive result was considered upon >2.0-fold increase in fluorescence with respect to untreated control.

### 2.12 Bioink preparation

The core bioink was produced by dissolving 2.5% (w/v) gelatin, 1.5% (w/v) UP-LVM alginate (Novamatrix; #4200201), and hyaluronic acid (3.0 mg/mL) in minimum essential medium (MEM, Gibco) without calcium. The solution was incubated for 2 h on a rotator at 37°C. The shell bioink was created with 3.5%(w/v) GelMa, 0.7% (w/v) gelatin, hyaluronic acid (3.0 mg/mL), 100 mM Ca^2+^, and 0.2% Lithium phenyl-2,4,6-trimethylbenzoylphosphinate (LAP) dissolved in serum-free, MEM with calcium (Gibco) in a 50 mL conical tube at 37°C for 12 h. The bioink was sterilized using a 0.45 µm syringe filter (09-719D; Fisherbrand). For bioink preparations containing dsECM, first dsECM was dissolved in MEM at a concentration of 0.1 mg/mL, followed by the above steps.

### 2.13 Coaxial bioprinting

For coaxial bioprinting, the core and shell bioinks were loaded into the integrated tissue organ printer (ITOP). The ITOP is made up of multiple cartridges to individually deliver cell-laden hydrogels, an XYZ stage/controller, dispensing module, and a closed chamber described previously (Jorgensen et al., 2020). A metal coaxial nozzle (core – 24Ga, shell – 18Ga) was used to print the bioinks at 60–90 KPa of air pressure. The bioprinter was used to deposit two layers of bioink to form a 1 × 1 cm construct in a 35 mm petri plate. Constructs were exposed to 10 s of UV to crosslink GelMa, followed by alginate crosslinking with 100 mM CaCl_2_ for 10 min. Crosslinked constructs were then submerged in cell culture media (DMEM) to wash out hyaluronic acid and gelatin. Medium was changed every other day.

### 2.14 Implantation of bioprinted constructs

Animal care, housing, and procedures were performed in accordance with the protocol approved by the Institutional Animal Care and Use Committee of Wake Forest School of Medicine. A total of three immunocompetent mice (8–9 weeks old, ∼40 g, male CD1 mice from Charles River Labs) were implanted with the cell-free coaxially bioprinted construct (core: 1.5% UP-LVM alginate and 0.1 mg/mL dsECM; shell: 3.5% GelMA and 0.1 mg/mL dsECM). Each mouse received two subcutaneous construct implantations (1 × 1 x ∼0.5 cm), one in each front shoulder behind the scapula. All mice were sacrificed after 7 days of implantation and the constructs were explanted for histology and immunostaining.

### 2.15 Histology

Sections of the wound samples were fixed for 48 h in 4% paraformaldehyde and then transferred to 70% ethanol before paraffin processing. A microtome (Leica) was used to generate 7 μm sections that were then stained with hematoxylin and eosin (Fischer et al., 2008) and Masson’s trichrome (Van De Vlekkert et al., 2020) for histological analysis and imaged by light microscopy.

### 2.16 Immunofluorescence staining

Immunostaining with antibodies against CD31 and α-SMA was used to visualize the vascularization of the implanted constructs. Slides were warmed for 30min at 58°C to ensure bonding to the slides. Antigen retrieval with proteinase K (Dako, Carpinteria, CA) was performed on all slides with incubation for 5min. Sections were then permeabilized for 10 min using 0.2% Triton X-100 in TBS (TBST) at room temperature. Nonspecific antibody binding was minimized by incubating sections for 10min in Protein Block Solution (Dako) at room temperature. Sections were incubated for 12 h at room temperature in a humidified chamber with primary antibodies for the following: CD31 (1:100 dilution; BS-195R; BiossUSA, Boston, MA) and α-SMA (1:1000 dilution; ab5694; Abcam, Cambridge, UK).

After the primary incubation, the slides were washed in Tris buffered saline (TBS) for 5 min thrice. Sections were then incubated for 60 min at room temperature in a humidity chamber with donkey anti-rabbit 488 (A-21206; Invitrogen) at a 1:500 dilution. After washing, sections were incubated with DAPI (D3571; Life Technologies, Eugene, OR) at a 1:10,000 dilution for 5min and then washed thrice in TBS for 5 min. Slides were cover slipped with VectaMount Aqueous Mounting Medium (H-5501; Vector Laboratories, Burlingame, CA). Negative controls were performed with secondary antibody incubations and a blocking solution incubation in place of the primary antibody. The negative controls demonstrated no immunoreactivity. All samples were imaged using a Leica DM4000B upright microscope with fluorescence at 647, 594, and 380 nm.

## 3 Results

### 3.1 *In vitro* biocompatibility of dsECM

A comprehensive evaluation of the cytotoxicity, hemocompatibility and immunocompatibility of the dsECM was performed following the American and European regulations with respect to nanosafety (ISO 10993). The type of assay, parameters tested and the selection criteria for each assay are summarized in Supplementary Table S1.

#### 3.1.1 Cytotoxicity

**FIGURE 1:**
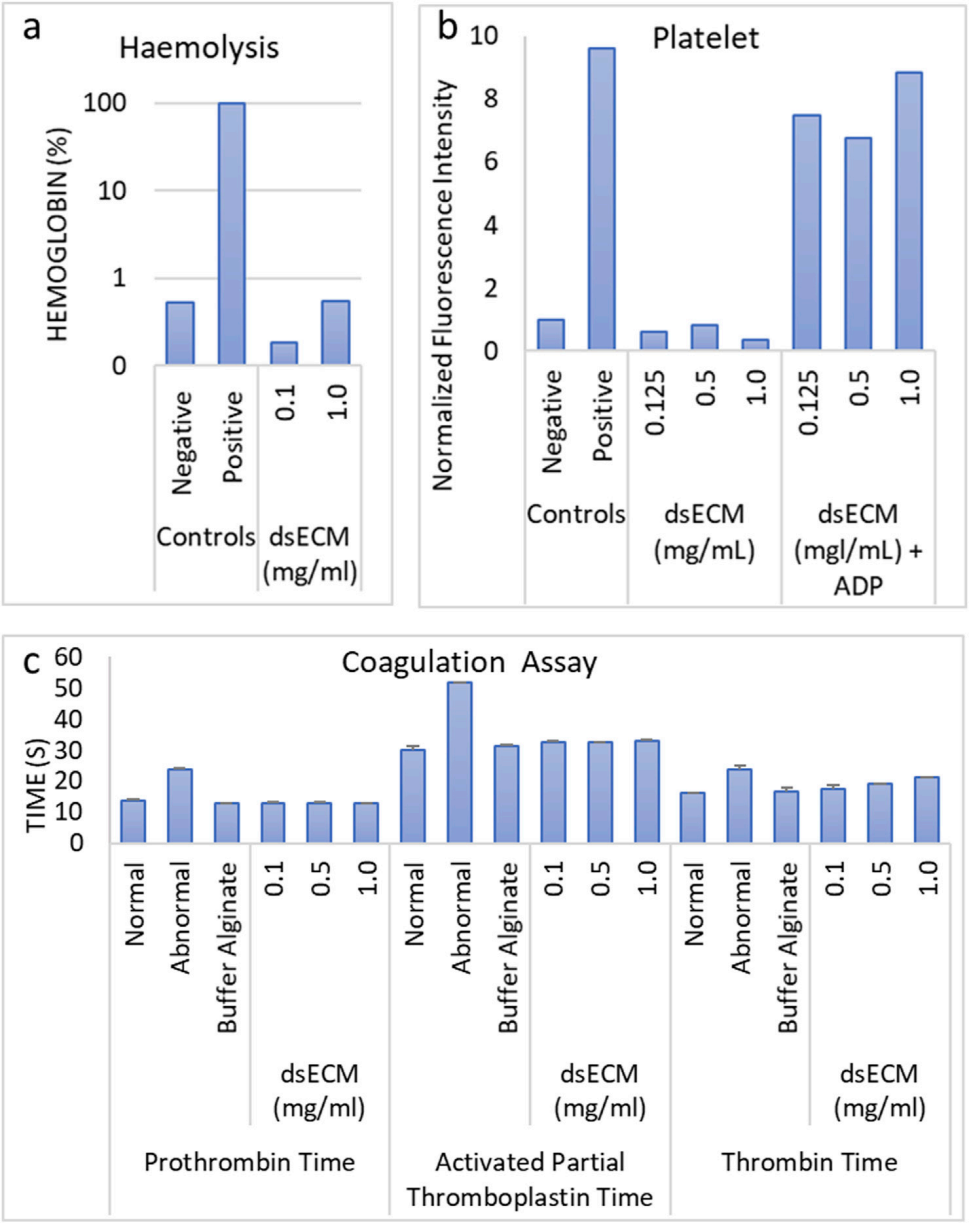
**(a)** The percentage of early and late apoptosis, necrotic and viable Jurkat cells, after a 6 h incubation with dsECM was assessed by flow cytometry, using a combination of Annexin V/Propidium Iodide (n = 3). Camptothecin (4 µg/mL) was used as a positive control. **(b)** A549, HEK293 and Jurkat cells were incubated with dsECM for 24 h and cell proliferation was measured by MTS assay (n = 3). The percentage of cell viability was normalized to cells without dsECM. **(c)** The content of intracellular reactive oxygen species (ROS) was analyzed by measuring the oxidation of the probe DCFH-DA in Jurkat and A549 cells, after incubation with dsECM for 4 h (n = 3). PMA (10 μM) was used as a positive control. Data are provided as mean with their standard deviation.

The cytotoxicity of the dsECM at concentrations of 0.125, 0.25, 0.5, 1.0 or 2.0 mg/mL was assessed using apoptosis, MTS and ROS assays. dsECM did not exhibit immediate toxic effects at any concentration, when tested with Jurkat cells. The percentage of viable cells was found to be >95% at all dsECM concentrations as shown in Figure 1a. MTS assay was performed on A549 (human alveolar adenocarcinoma), HEK293 (human embryonic kidneyand) and Jurkat (immortalized human T lymphocyte) cells after 24 h incubation with dsECM. The percentage cell viability normalized to control cells (without dsECM) is presented in Figure 1b. A549 cells did not display a significant change in viability at any concentration of dsECM. However, there was a sharp decrease in the viability of HEK293 cells (62%), at a concentration of 2.0 mg/mL. Jurkat cells also displayed a concentration-dependent decrease in viability, with only 69% being viable at 2.0 mg/mL of dsECM. The content of intracellular ROS was analyzed by measuring the oxidation of DCFH-DA (2′, 7-dichlorofluorescein diacetate) in A549 and Jurkat cells by flow cytometry. ROS content from both the cell lines was found to be within the acceptable range of <1.4-times the negative control (untreated cells) at every dsECM concentration tested, except at 2.0 mg/mL for Jurkat cells (2.1 times; Figure 1c).

#### 3.1.2 Hemocompatibility

**FIGURE 2:**
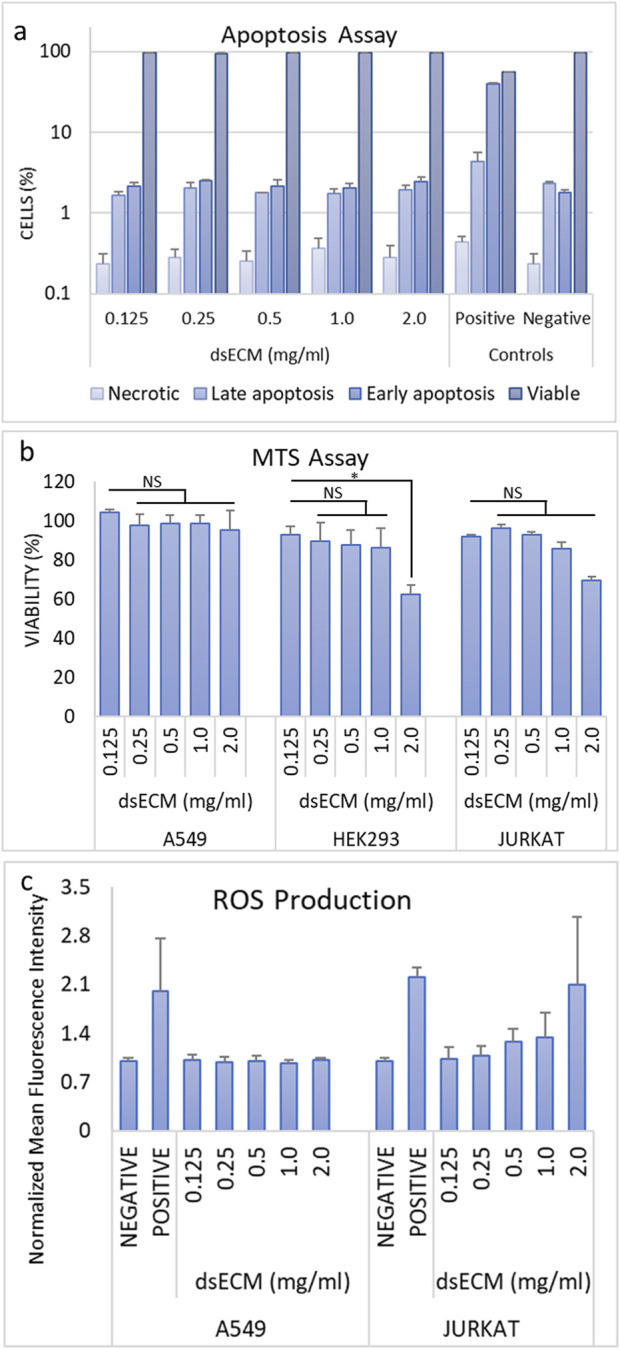
**(a)** The hemolysis was assessed by colorimetric quantification of hemoglobin in whole-blood, upon exposure to dsECM (n = 2 human donors; pooled). **(b)** Platelet activation was determined by the quantification of CD62P expression on the surface of activated platelets, by flow cytometry (n = 2 human donors; pooled). Adenosine diphosphate (ADP; 20 µM) was used as positive control. **(c)** Changes in the intrinsic, extrinsic and common coagulation pathways due to the binding or depletion of coagulation factors with dsECM were evaluated by measuring the time necessary (in seconds) for clot formation. A pool of human plasma from at least three donors was used and clot formation was detected by a viscosity-based detection system. Normal and pathological plasma were used as internal controls. Data are provided as mean with their standard deviation.

Based on cytotoxicity results, dsECM concentrations ranging from 1.0 to 0.1 mg/mL were used for further testing. Hemocompatibility was evaluated by performing hemolysis, thrombogenecity and coagulation, and platelet activation assays. The criteria for hemolysis according to the Standard Practice for Assessment of Hemolytic Properties of Materials (ASTM E2524-08) (Barry et al., 2024) is mentioned in Supplementary Table S1. In brief, a test material is considered to not cause damage to red blood cells (RBCs) and is regarded as non-hemolytic if hemoglobin value is <2%. The dsECM did not induce hemolysis (hemoglobin <2%) in human erythrocytes at both concentrations, however hemolysis was found to be higher at 1.0 mg/mL (0.55%) than 0.1 mg/mL (0.18%) (Figure 2a). To determine platelet activation, the expression of CD62P on the surface of activated platelets was measured by flow cytometry (Figure 2b). Adenosine diphosphate (ADP) was used as a positive control as it is released from the dense granules during platelet activation. The fluorescence increase was found to be < 2-times with respect to the negative control, which suggested that dsECM did not induce platelet activation at any tested concentration. The time taken by clotting factors for clot formation through the intrinsic, extrinsic and common coagulation pathways were assessed by partial thromboplastin time (APTT), prothrombin time (PT) and thrombin time (TT) tests, respectively. Both TT and APTT were found to be higher than negative control (normal) for all dsECM concentrations (Figure 2c). Moreover, TT exhibited a concentration-dependent increase, with its value being slightly above the physiological value (21.05 ± 0.35 s) at 1.0 mg/mL of dsECM. However, there was no physiologically significant prolongation of coagulation (>2-times) at any tested concentration of dsECM.

#### 3.1.3 Imunocompatibility

**FIGURE 3:**
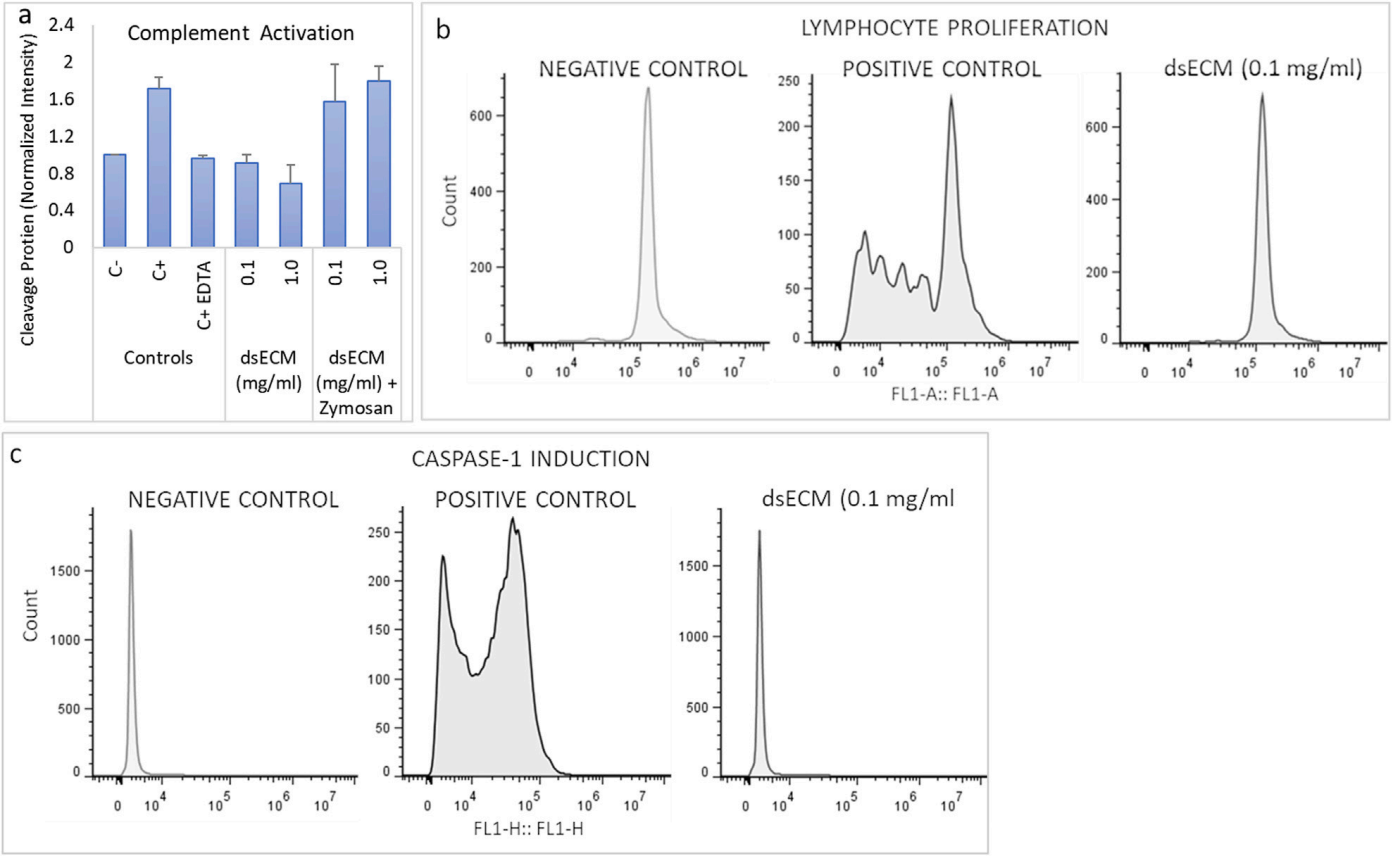
**(a)** Complement system activation was assessed by analyzing the degradation of C3 factor by western blot, after incubation of human plasma with dsECM for 1 h. Zymosan A (1 mg/mL) and EDTA (10 mM) were used as positive and reversion reaction control, respectively. The band intensity corresponding to C3b fraction was compared to the basal level of C3b (negative control). **(b)** PBMCs stained with CFSE were cultured with and without dsECM for 7 days and their proliferation rate was determined by flow cytometry. Phytohemagglutinin (PHA-M; 10 µg/mL) was used as a positive control, being a recognized polyclonal T-cell mitogen (n = 3 human donors; pooled). **(c)** Caspase-1 activity was determined by flow cytometry, using a fluorescent inhibitor probe to label active caspase-1 enzyme in THP-1 cells, following 5 h of incubation with dsECM. Camptothecin (4 µg/mL) was used as positive control. Data are provided as mean with their standard deviation.

**FIGURE 4:**
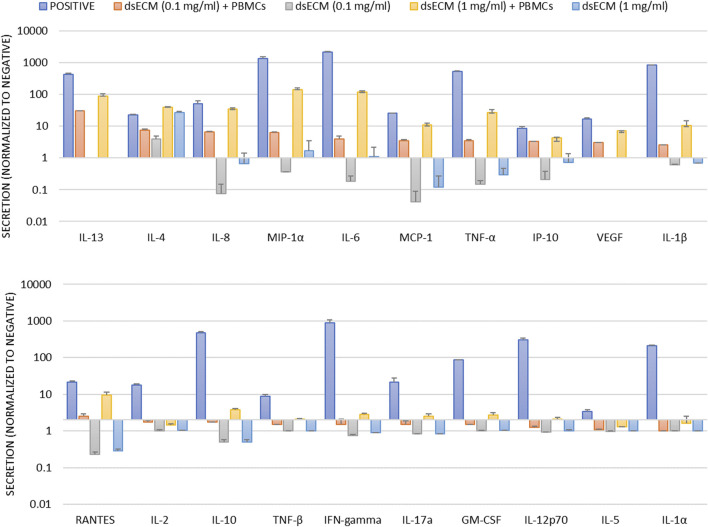
Human PBMCs isolated from blood sample pool of three healthy donors were incubated for 24 h with dsECM. PHA-M (10 µg/mL) and LPS (1 µg/mL) were used as positive controls. Following incubation, the supernatant was used for cytokine quantification by a Luminex magnetic bead panel. The concentration of each cytokine was determined by the individual standard curve. A positive result is considered upon increase of fluorescence of >2.0-fold respect to untreated control. Data are provided as mean with their standard deviation.

The imunocompatibility of dsECM was assessed based on complement activation, leukocyte proliferation, caspase-1 activation and inflammatory cytokine production. Complement activation results in the degradation of C3 factor by C3-convertase that can be analyzed by Western Blot. Quantitative comparison of bands corresponding to C3b fraction with respect to basal C3b level showed that dsECM did not induce activation of the complement system, at any tested concentration (0.91 ± 0.08 for 0.1 mg/mL dsECM and 0.69 ± 0.19 for 1.0 mg/mL dsECM; Figure 3a). The ability of lymphocytes to undergo clonal proliferation upon stimulation with 0.1 mg/mL dsECM was also assessed. The dsECM did not stimulate lymphocyte clonal expansion, while five cellular divisions were observed with phytohemagglutinin as a positive control of proliferation (Figure 3b). Caspase-1 activation was evaluated by flow cytometry, using a fluorescent inhibitor probe to label active intracellular caspase-1 enzyme in THP-1 cells (human monocytic cell line). As shown in Figure 3c, incubation with 0.1 mg/mL dsECM did not induce activation of the Caspase-1 pathway, while a fluorescent signal was observed with cells incubated with Camptothecin as a positive control. Figure 4 shows the relative concentration (normalized to basal level) of cytokines produced by PBMCs, after 24 h incubation in media supplemented with dsECM at 0.1 and 1.0 mg/mL. Cytokine secretion was found to be dependent on dsECM concentration, with all cytokines having a higher concentration at 1.0 mg/mL compared to 0.1 mg/mL, except IL-2. Results were deemed significant when an increase of >2.0-fold was observed, compared to the negative control. dsECM (0.1 mg/mL) induced the production of anti-inflammatory cytokines (IL-4, IL-13), pro-inflammatory cytokines (IL-1β, IL-6 and TNF-α), chemokines (MCP-1, IP-10, MIP-1α, IL-8 and RANTES) and VEGF, while production of all the cytokines except IL-1α, IL-2 and IL-5 was induced at dsECM concentration of 1.0 mg/mL.

### 3.2 3D bioprinting and *in vivo* biocompatibility of dsECM

**FIGURE 5:**
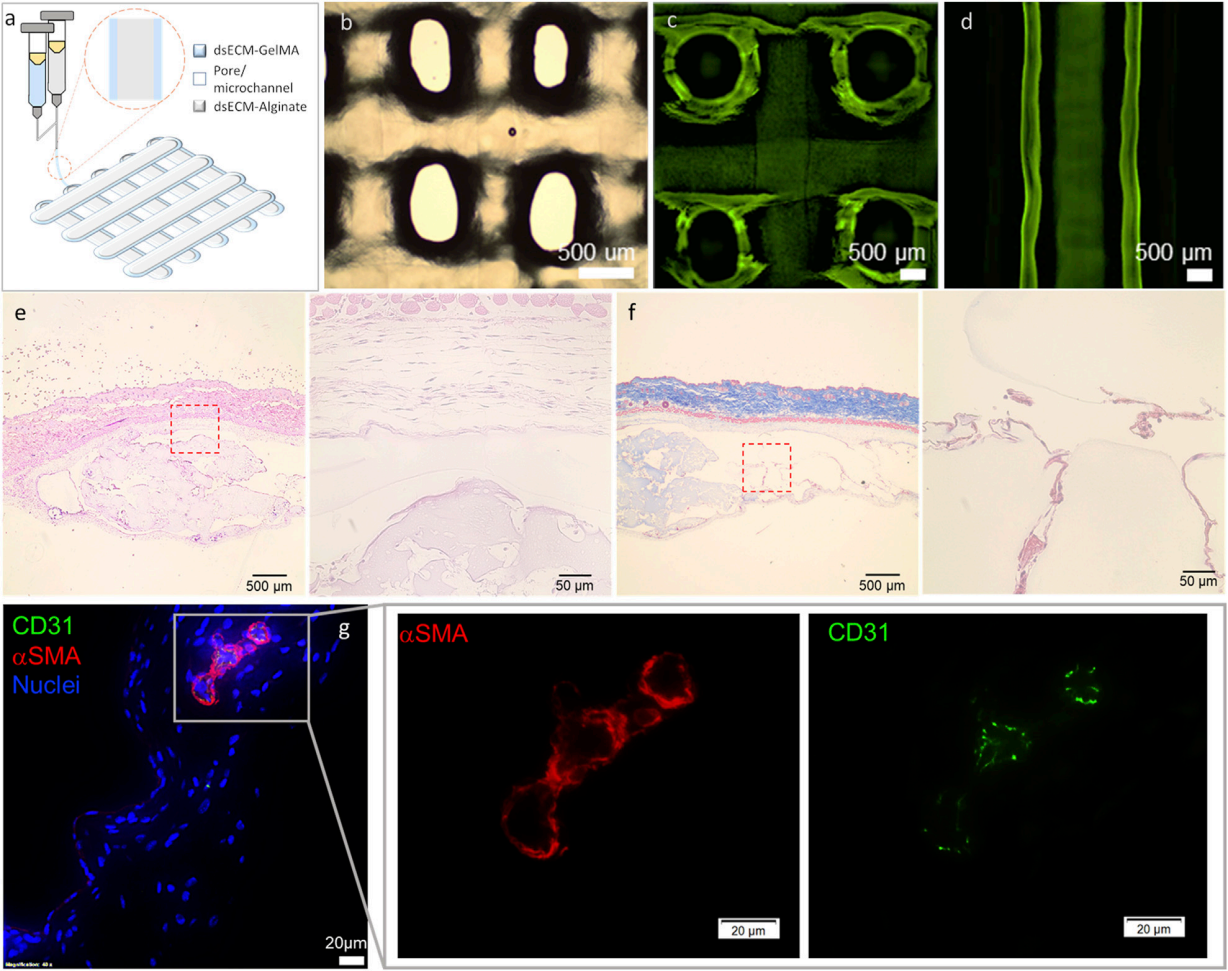
**(a)** Schematic and **(b)** gross appearance of the coaxially bioprinted construct. **(c,d)** Printing with FITC-dextran (250 kDa) labelled core bioink showed high printability, printed strut/fiber continuity and distinct regional localization of core and shell. Representative histological images of the retrieved coaxially printed constructs - **(e)** H&E and **(f)** Masson’s Trichrome at low ang high magnification. **(g)** Representative immunofluorescence images stained for CD31 (green), α-smooth muscle actin (αSMA, red), and DAPI (blue) at low ang high magnification. The coaxially printed constructs (cell-free; 1.5% UP-LVM-dsECM_0.1 core and 3.5% GelMA-dsECM_0.1 shell) were retrieved at 7 days after implantation in CD1 immune-competent mice.

We had previously demonstrated that alginate (1.5% UP-LVM) capsules supplemented with dsECM provided essential biochemical cues to preserve human islet functionality during long-term *in vitro* culture (Asthana et al., 2023). Therefore, the next step was to determine the adaptability of dsECM-alginate as a bioink, to enable 3D bioprinting of complex human pancreatic tissues. Towards this, a coaxial (core-shell) bioprinting strategy was adopted, with a core of dsECM-alginate, surrounded by a shell of dsECM-GelMA (gelatin methacrylate) bioink (Figure 5a). 1.5% UP-LVM alginate had previously been used for islet encapsulation (Asthana et al., 2023), however, it could not be adapted directly as a bioink owing to its low viscosity. Therefore, additives such as gelatin and hyaluronic acid (HA) were added to alginate and GelMA, which allowed modification of bioink viscoelastic properties for the bioprinting process, but would be later washed out in culture. Thereafter, a construct with a 1.5% UP-LVM alginate core bioink and 3.5% GelMA shell bioink was coaxially printed, using the optimized bioink and printing parameters (Figure 5b). The construct showed high printability, with the printed struts/fibers maintaining continuity and uniform diameters throughout the construct as well as distinct regional localization and containment of green fluorescence-labeled alginate core (Figure 5c, d). dsECM (0.1 mg/mL) was seamlessly incorporated in both the core and shell bioinks, without any change in printing process parameters. A cell-free construct was coaxially bioprinted (core: 1.5% UP-LVM alginate and dsECM_0.1; shell: 3.5% GelMA and dsECM_0.1) and implanted in the subcutaneous site of CD1 mice (n = 3). After 7 days of implantation, the construct was explanted to evaluate the early phase host inflammatory response to the composite materials. Histological analysis indicated minimal encapsulation of the implanted constructs, suggesting negligible early inflammatory response, while host cells were allowed to infiltrate into the microchannel structure (Figure 5e, f). Immunofluorescence analysis of CD31^+^ and α-SMA expression indicated vascularization of the implanted constructs (Figure 5g), as early as 7 days post-implantation, in the poorly-vascularized subcutaneous site.

## 4 Discussion

Classical detergent- and perfusion-based whole organ decellularization can present obstacles, such as maintenance of sterility and low endotoxin levels, and the requirement for large volumes of decellularization reagents that can impede scale-up and transfer to a GLP/GMP facility. The decellularization protocol employed here, avoids the use of both chemical detergents and perfusion-based systems, thereby presenting an efficient and innovative alternative to overcome these impediments and enable seamless translation. However, as shown by our previously conducted proteomic characterization, this method cannot completely remove plasma- or vesicle membrane-bound proteins (found to be enriched, especially in dsECM) (Asthana et al., 2021). It has been suggested that cellular remnants in decellularized biomaterials might be cytotoxic or elicit unfavorable responses upon *in vivo* transplantation (Cravedi et al., 2017). Therefore, it was imperative to perform an extensive characterization to determine the biocompatibility and safety of dsECM and also establish a certain concentration or a range that would be compatible with cells.

The test for cell viability and determination of cytotoxic effects related to cell death and ROS content are the first evidence suggesting potential toxicity of a biomaterial. dsECM did not exhibit any immediate toxic effects (Figure 2a), however there was significant reduction in viability following prolonged incubation and ROS content at the highest concentration tested (2.0 mg/mL) (Figure 2b, c). Therefore, it was considered cytotoxic at this concentration and a range of 0.1–1.0 mg/mL was chosen for further testing. The hemocompatibility of dsECM was tested for evaluating its interaction with erythrocytes, platelets and coagulation factors and determination of any acute *in vitro* hemolytic properties. The hemolysis assay was performed in accordance to the criteria in the Standard Practice for Assessment of Hemolytic Properties of Materials (Barry et al., 2024), which states that a hemolysis percentage higher than 5% indicates that the test material can cause damage to the red blood cells. Platelet activation was also assessed by flow cytometry for the expression of CD62P, a platelet-specific selectin protein which is expressed on the surface of activated platelets. Adenosine diphosphate (ADP) was used as a positive control since it is released from the dense granules during platelet activation. Overall, dsECM did not exhibit any hemolytic properties (Figure 2a) nor induce platelet activation (Figure 2b) at the tested concentrations. The dsECM was also tested for alteration of coagulation. Although, a minor increase in TT was observed, there was no physiologically significant prolongation of coagulation (>2-times) at any tested concentration of dsECM (Figure 2c). These results provide support to the safety of the biomaterial if it comes in contact with the host cells, upon implantation.

The induction of immunological responses was evaluated by complement activation, leukocyte proliferation, caspase-1 activation and cytokine profile. The complement system plays a critical role in the defense against infection, clearance of apoptotic cells and immune complexes and also contributes to the coordination of adaptive immune response. The clinical relevance of complement activation is to correlate it with Complement Activation Related Pseudo Allergy (CARPA), whose mechanism does not involve IgE (Szebeni, 2014). A lymphocyte proliferation assay was also performed to evaluate the ability of lymphocytes to undergo clonal proliferation when stimulated *in vitro* by dsECM. Overall, dsECM did not result in the induction of the complement cascade (Figure 3a) or clonal proliferation of lymphocytes (Figure 3b) *in vitro*. Inflammasomes are multimeric protein complexes that promote caspase-1 activation and the secretion of pro-inflammatory cytokines IL-1beta and IL-18, triggering a rapid and pro-inflammatory form of cell death called pyroptosis (Broz and Dixit, 2016). Results indicated that the dsECM did not induce caspase-1 activation pathway at the concentration tested (Figure 3c), again highlighting its safety as a biomaterial. An evidence for the biological functionality of dsECM is the stimulation of cytokines and chemokines after incubation with PBMCs (Figure 4). At 0.1 mg/mL, the dsECM induced the production of IL-13, which is known to participate in dsECM remodeling as a profibrotic interleukin. The induction of VEGF was also observed, this could have a role in immunity, inflammation and in the angiogenetic property of the dsECM (Llacua et al., 2018).

Following comprehensive proteomic (Asthana et al., 2021), functional (Tamburrini et al., 2020; Asthana et al., 2023) and *in vitro* biocompatibility characterization of dsECM, the next step was to develop dsECM-based bioinks for 3D bioprinting and automated scale-up of construct biofabrication for islet transplantation. Herein, a coaxial bioprinting approach was adopted, with a core of alginate-dsECM, surrounded by a shell of dsECM-GelMA. The alginate-dsECM core can provide immune protection to the islets, along with a peri-islet niche, while the biodegradable GelMA shell would be permissive to cell migration and remodeling, to promote construct integration and increase biocompatibility. Further, supplementing the shell bioink with dsECM could provide additional growth factors, acting as chemo-attractants for accelerated vascularization that could result in higher integration with host tissue and prevention of fibrotic encapsulation. The bioprinted construct was implanted in the subcutaneous space as it is less invasive and can allow construct retrieval in case of an adverse event. As dsECM exists in a lyophilized powder form as opposed to dECM (hydrogel), it allowed seamless incorporation in existing bioinks. For previous functional studies (Tamburrini et al., 2020; Asthana et al., 2023), we had successfully used dsECM-1.5% UP-LVM for human islet encapsulation, however, 1.5% UP-LVM had never been successfully bioprinted due it its very low viscosity. Using our bioink development method, we were able to successfully bioprint 1.5% UP-LVM and that too with a coaxial nozzle along with a GelMA based-shell, making this study the first instance of bioprinting alginate at this concentration. This bioink development approach can potentially be used to enable bioprinting of other difficult to print low-viscosity hydrogels.

In preliminary *in vivo* implantation experiments, the dsECM-alginate/dsECM-GelMA construct resulted in minimal early phase inflammatory host response, as evident from the absence of foreign body giant cells, while supportive host cells were observed infiltrating into the microchannel structure (Figure 5e). Although fibrosis can take longer to develop, we did not observe demonstrable early signs of inflammatory response during the 7-day implantation of the construct. It has been shown that early-phase immune response can be representative of long term biocompatibility (Wang et al., 2017), however, a follow-up long-term animal study is needed to establish the biocompatibility of the composite hydrogel. Moreover, the presence of CD31+/αSMA+ cells within this short implantation period, indicates the angiogenic potential of our bioprinted construct (Figure 5g). The lag period between implantation and integration with the host vasculature can result in early tissue necrosis due to insufficient oxygen supply, especially for hypoxia-sensitive islets. Therefore, these early signs of microvascular growth could be of great significance towards revascularization of islet grafts. This was a pilot observational study with the purpose of precluding a catastrophic failure of the composite materials due to an early immune response. These results provide a go/no go decision before embarking upon a long-term biocompatibility study including multiple conditions and time points. However, the lack of a negative control in the implantation experiments represents a limitation of this study. Future work will include such controls to more comprehensively evaluate the performance of the implant and its integration with host tissue.

Overall, a working range of dsECM concentration was determined in which it was found to be biocompatible, and should provide a safe starting concentration for *in vivo* applications where it can come in contact with multiple cell types. More importantly, as an alternative to harsh traditional decellularization methods, we have established a novel gentler approach that produces a functional and biocompatible biomaterial. Lastly, we have also demonstrated that the dsECM powder can be seamlessly incorporated in conventional hydrogels to transform them into human pancreas-specific bioinks that also suggest vascularization properties.

## Data Availability

The raw data supporting the conclusions of this article will be made available by the authors, without undue reservation.
