# Supplementary material for: Comprehensive biocompatibility profiling of human pancreas-derived biomaterial
Source: Front Bioeng Biotechnol. 2025 Apr 25;13:1518665. doi: 10.3389/fbioe.2025.1518665 (PMC12062020; doi:10.3389/fbioe.2025.1518665)
Supplement: Supplementary file 1 [file DataSheet1.pdf]

Supplementary Table S1. Parameters for testing *in vitro* safety and biocompatibility

	In vitro Assay	Test Type	Parameter measured	Biocompatibility/Safety Criteria
Cytotoxicity	MTS [1, 2]	Colorimetric	Number of living cells due to the reduction of the MTS reagent by dehydrogenase enzymes in metabolically active cells.	> 70% viable cells
	Reactive Oxygen Species (ROS) production [3]	Fluorescence	Presence of reactive oxigen species (ROS) using dichlorofluorescein diacetate (DCFH-DA). The intracellular deacetylation of DCFH-DA and the oxidation of DCFH by ROS, turns it into a fluorescent compound that is measured by flow cytometry.	The relative fluorescent intensity is not > 1.4 with respect to untreated control
	Apoptosis [1, 2]	Fluorescence	Loss of plasma membrane integrity due to exposition of phosphatidylserine (PS) on cells undergoing apoptosis and permeability of the membrane on necrotic cells allowing propidium iodide (PI) to pass through. Protein Annexin V binds with high affinity to PS. Fluorochrome-conjugated Annexin V and PI are used to detect apoptotic cells by flow cytometry.	Valid if > 80% viable cells<br>**Viable**: Annexin V negative, PI negative.<br>**Early apoptosis**: Annexin V positive, PI negative.<br>**Late apoptosis**: Annexin V positive, PI positive.<br>**Necrotic**: Annexin V negative, PI positive.
Hemocompatibility	Hemolysis [4]	Colorimetric	Hemoglobin released from damaged erythrocytes using cyanmethemoglobin (CMH) reagent	**Non hemolytic**: < 2%<br>**Slightly hemolytic**: 2-5%<br>**Hemolytic**: > 5%
	Platelet activation [5, 6]	Fluorescence	Expression of CD62P on the surface of the activated platelet by flow cytometry. Aggregation is also checked under microscope after Wright's stain.	The relative fluorescent intensity is not > 2.0 with respect to untreated control
	Coagulation [7]	Clot formation	The time taken by plasma proteins (clotting factors) for clot formation through the intrinsic, extrinsic and common coagulation pathways. Each pathway can be assessed by a specialized test: partial	**APTT** ≤ 34.1 sec<br>**PT** ≤ 13.4 sec<br>**TT** ≤ 21 sec<br>**Prolongation** ≥ 2-fold versus untreated control is considered physiologically significant

			thromboplastin time (APTT) prothrombin time (PT) and Thrombin time (TT), respectively. Prolongation of coagulation times suggests that the biomaterial either depletes or inhibits clotting factors.	
Immunocompatibility	Complement activation [8]	Immunoblot	Identification of C3b degradation factor as a result of the C3 convertase activation.	< 2 times with respect to basal level of C3b
	Leukocyte proliferation [9]	Fluorescence	Clonal proliferation of peripheral blood mononuclear cells (PBMCs) using CFSE (carboxyfluorescein diacetate succinimidyl ester) and measured by flow cytometry.	No distribution of fluorescent-CFSE to daughter cells
	Caspase 1 activation [10]	Fluorescence	Inflammation response in living cells using a fluorescent inhibitor probe which enters each cell and irreversibly binds to activated caspase-1	Cells have no green fluorescent signal
	Inflammatory cytokines [11]	Fluorescence	Inflammatory cytokine profile of peripheral blood mononuclear cells (PBMC) measured by flow cytometry	The concentration of each cytokine determined by the individual standard curve is not >2.0-fold with respect to untreated control

References

[1] ISO 10993-5, Biological evaluation of medical devices: Part 5, Tests for in vitro cytotoxicity. https://www.iso.org/standard/36406.html

[2] ASTM F1903 – 98, Standard Practice for Testing for Biological Responses to Particles in vitro. https://www.astm.org/f1903-98r03.html.

[3] Z.B. Stern ST, NCL Method GTA-7: Hepatocyte Primary ROS Assay. https://ncl.cancer.gov/resources/assay-cascade-protocols DOI: 10.17917/0WF6-GX40.

[4] ASTM F 756-00. Assessment of hemolytic properties of materials. https://www.astm.org/f0756-17.html.

[5] L.C. Burzynski, N. Pugh, M.C.H. Clarke, Platelet Isolation and Activation Assays, Bio Protoc 9(20) (2019) e3405.

[6] E. Fröhlich, Action of Nanoparticles on Platelet Activation and Plasmatic Coagulation, Curr Med Chem 23(5) (2016) 408-30.

[7] P.T. Cedrone E, Neun BW, Dobrovolskaia MA, NCL Method ITA-12: In vitro Analysis of Nanoparticle Effects on Plasma Coagulation Time. https://ncl.cancer.gov/resources/assaycascade-protocols DOI: 10.17917/7SF3-7A88.

[8] C.E. Neun BW, Dobrovlskaia MA, NCL Method ITA-5.1: Qualitative Analysis of Total complement Activtion by Western Blot. https://ncl.cacner.gov/resources/assay-cascade-protocols DOI: 10.17917/HA0Q-5B49.
[9] L.M. Muul, G. Heine, C. Silvin, S.P. James, F. Candotti, A. Radbruch, M. Worm, Measurement of proliferative responses of cultured lymphocytes, Curr Protoc Immunol Chapter 7 (2011) Unit7 10.
[10] A. Sokolovska, C.E. Becker, W.K. Ip, V.A. Rathinam, M. Brudner, N. Paquette, A. Tanne, S.K. Vanaja, K.J. Moore, K.A. Fitzgerald, A. Lacy-Hulbert, L.M. Stuart, Activation of caspase-1 by the NLRP3 inflammasome regulates the NADPH oxidase NOX2 to control phagosome function, Nat Immunol 14(6) (2013) 543-53.
[11] P.T. Cedrone E, Neun BW, Tyler A, Dobrovolskaia MA, NCL Method ITA-27: Multiplex Enzyme-Linked Immunosorbent Assay (ELISA) for Detection of Human Cytokines in Culture Supernatants. https://ncl.cancer.gov/resources/assay-cascade-protocols DOI:10.17917/P2T3-8P84.
